# The Applicability of AWaRe-Based Antibiotic Quality Indicators to Assess the Appropriateness of Antibiotic Prescribing in Primary Healthcare in South Africa: A Multicentre Point Prevalence Study and Implications for the Future

**DOI:** 10.3390/antibiotics15060562

**Published:** 2026-06-01

**Authors:** Audrey K. Chigome, Aislinn Cook, Yasmina Johnson, Sabiha Essack, Adrian Brink, Marc Mendelson, Stephen M. Campbell, Brian Godman, Johanna C. Meyer

**Affiliations:** 1 Department of Public Health Pharmacy and Management, School of Pharmacy, Sefako Makgatho Health Sciences University, Ga-Rankuwa, Pretoria 0208, South Africa; stephen.campbell@smu.ac.za (S.M.C.); hannelie.meyer@smu.ac.za (J.C.M.); 2 South African Vaccination and Immunization Centre, Sefako Makgatho Health Sciences University, Ga-Rankuwa, Pretoria 0208, South Africa; 3 Antibiotic Policy Group, Institute for Infection and Immunity, City St. George’s, University of London, London SW17 0RE, UK; aicook@citystgeorges.ac.uk; 4 Nuffield Department of Primary Care Health Sciences, University of Oxford, Oxford OX2 6GG, UK; 5 Pharmaceutical Services, Medicine Management, Laboratory and Blood Services Support, Western Cape Department of Health and Wellness, Cape Town 8001, South Africa; yasmina.johnson@westerncape.gov.za; 6 School of Pharmacy, University of the Western Cape, Cape Town 7535, South Africa; 7 Antimicrobial Research Unit, School of Health Sciences, University of KwaZulu-Natal, Durban 3629, South Africa; essacks@ukzn.ac.za; 8 Division of Medical Microbiology, Faculty of Health Sciences, University of Cape Town, Cape Town 7700, South Africa; adrian.brink@uct.ac.za; 9 National Health Laboratory Service, Groote Schuur Hospital, Cape Town 7925, South Africa; 10 Institute of Infectious Disease and Molecular Medicine, Faculty of Health Sciences, University of Cape Town, Cape Town 7700, South Africa; 11 Division of Infectious Diseases and HIV Medicine, Department of Medicine, Groote Schuur Hospital, University of Cape Town, Cape Town 7700, South Africa; marc.mendelson@uct.ac.za; 12 School of Health Sciences, University of Manchester, Manchester M13 9PL, UK

**Keywords:** antibiotic prescribing, antibiotic stewardship, antimicrobial resistance, applicability, AWaRe, clinimetric properties, low- and middle-income countries, primary healthcare, quality indicators, respiratory tract infections, South Africa

## Abstract

**Background/Objectives**: Inappropriate antibiotic prescribing in primary healthcare (PHC) contributes to bacterial antimicrobial resistance (AMR). Antibiotic stewardship, including measuring the appropriateness of antibiotic prescribing using quality indicators, is a priority in PHC where most antibiotics are used. Using previously developed WHO AWaRe classification-based quality indicators, we aim to test the clinimetric properties of 13 acute respiratory tract infection (RTI) quality indicators and qualitatively explore factors influencing PHC antibiotic prescribing in South Africa. **Methods**: We conducted a mixed-methods exploratory feasibility study using point prevalence surveys (PPSs) with clinimetric assessment and prescriber interviews. PPSs were conducted at four PHC facilities in Gauteng province that had taken part in previous PPS studies, alongside face-to-face interviews with PHC personnel. **Results**: In total, 52/52 (100%) RTI patients received antibiotics. Four (30.8%) indicators achieved scores above 85%, while six (46.2%) scored below 50%. All indicators had applicability scores ≥10%. Twelve (92.3%) indicators had a measurability score of 100%, while one (7.7%) had a measurability score <75%. Twelve (92.3%) indicators met all predefined acceptable scores for applicability and measurability. No participant knew of the WHO’s AWaRe classification and none had specific training on antibiotic prescribing and AMR. They also had no antibiotic stewardship programmes (ASPs) or specific antibiotic-prescribing guidelines at their facilities. Key factors affecting antibiotic prescribing included shortages, patient expectations and fear of complications. **Conclusions**: The indicators demonstrated acceptable clinimetric properties in South Africa. Robust locally validated indicators, combined with ASPs promoting the AWaRe classification, are imperative for accurate assessment and improvement of antibiotic prescribing.

## 1. Introduction

There are continuing high levels of inappropriate antibiotic prescribing in primary healthcare (PHC) among low- and middle-income countries (LMICs), including African countries, contributing to persistent high levels of bacterial antimicrobial resistance (AMR) [1,2,3,4,5,6]. Since 80–90% of antibiotics used for the treatment of infectious diseases are prescribed in primary care settings, improving antibiotic-prescribing practices in this sector, including greater use of Access antibiotics, is critical for safeguarding the effectiveness of existing antibiotics [4,7,8,9,10].

In South Africa, there is continuing concern regarding inappropriate antibiotic prescribing, which includes high levels of prescribing for viral infections such as upper respiratory tract infections (URTIs) [11,12,13,14,15]. Alongside this, there is concern over the increasing use of Watch antibiotics, with both concerns exacerbated by variable compliance with current treatment guidelines [15,16,17]. This needs to be urgently addressed, with high levels of Watch and Reserve antibiotics contributing to AMR [8,9,18,19]. However, there are ongoing challenges with improving the use of antibiotics in South Africa, mainly due to the sub-optimal implementation of antimicrobial stewardship (AMS) initiatives in primary care [15,20]. In view of these concerns, AMS activities, including measuring the quality of antibiotic prescribing in primary care as part of planned antimicrobial stewardship programs (ASPs), must be prioritized in South Africa when the National AMR Strategy Framework is eventually updated [20,21].

We are aware that quality indicators have been widely developed as AMS tools in high-income countries to measure, monitor and improve the quality of antibiotic prescribing across settings [15,22,23,24,25,26,27]. Additionally, most available quality indicators do not currently reflect the guidance in the World Health Organization (WHO)’s Access, Watch, Reserve (AWaRe) antibiotic book for managing a range of bacterial infections commonly presenting in primary care [27,28,29]. This is important, with concerns regarding the robustness of current antibiotic guidelines among LMICs [30]. These gaps constrain the development of evidence-informed policies and hinder the design and implementation of context-appropriate quality-improvement strategies to address persistent high AMR rates. This includes concerns over addressing continuing non-adherence to national antibiotic guidelines among LMICs, including among African countries [9,12,15,16,18,31,32]. Improved access to robust guidelines, including via Apps, alongside updates and any adaptation of the WHO AWaRe guidance based on local resistance patterns, are a key part of future initiatives to reduce AMR among LMICs [18,33,34,35].

We previously documented that researchers at City St Georges, University of London (SGUL), United Kingdom, have developed a global model set of quality indicators based on the WHO AWaRe antibiotic book, which incorporates both primary and hospital care as well as general indicators, to improve future antibiotic use [27]. These indicators can then be adapted locally within individual LMICs to improve their acceptance and utility [27]. We subsequently adapted the model global quality indicators alongside adding new indicators relevant to the specific contexts in South Africa, using the RAND/UCLA Appropriateness Method (RAM). This resulted in a final set of 61 quality indicators to specifically assess the quality and appropriateness of antibiotic prescribing among public PHC facilities in South Africa [15].

However, the successful adoption and implementation of any pertinent quality indicator in primary care in South Africa will depend not only on the validated systematic consensus method used to develop it but also on the robustness of its clinimetric or measurement properties to ensure accurate quality assessment and, hence, track improvement [24,36,37,38]. Clinimetric properties include the applicability and measurability of any potential quality indicator. This is important for determining whether their implementation accurately and consistently measures what they are intended to measure [24,36,39]. In view of this, following the development of quality indicators in primary care targeting LMICs starting with South Africa [15], testing them prior to implementation is essential for refining and subsequently implementing only those indicators that are valid, measurable and applicable to improve future antibiotic use in practice among PHC facilities in South Africa [15,27,36].

As a result, the objectives of the study reported in this paper were three-fold. First, we aimed to test the clinimetric properties of a subset of 13 of the 61 recently developed AWaRe based quality indicators and indicators specific to the South African context, focusing on respiratory tract infections (RTIs) [36]. Second, assess the appropriateness of antibiotic prescribing in PHC public sector facilities in South Africa using data from point prevalence surveys (PPSs) [15,40]. The final objective was to explore key factors that could influence current antibiotic-prescribing practices and the applicability of quality indicators in the South African public sector context. We believe this comprehensive approach, which includes empirical data and an indicator performance assessment, coupled with insights from PHC prescribers, will help improve future antibiotic prescribing not only in South Africa but also in other LMICs.

The rationale for selecting the subset of 13 indicators addressing RTIs, specifically for the first objective, was because approximately 50% of the infection-specific indicators pertain to RTIs globally and in South Africa [41], and RTIs are typically the most common presentations in patients seeking care for their infectious disease in South Africa [12,13,14,15]. Furthermore, from a South African perspective, the focus on RTIs is also important as there have been high levels of inappropriate prescribing of antibiotics for patients with RTIs among PHC facilities in the country [12,13,14,15].

## 2. Results

### 2.1. PPS Results

Overall, 121 patients were considered eligible for the PPS, meaning they were consulting for acute symptoms at the four participating public sector facilities on the days of data collection. Among them, 96 (96/121; 79.3%) patients received at least one antibiotic for acute infection symptoms during the PPS. Fifty-two patients (52/121; 43%) presented with at least one acute RTI-related symptom and they all received at least one antibiotic. Only these 52 patients were included in the testing of the quality indicators’ clinimetric properties in this study (Supplementary Figure S1).

The majority of patients were female (*n* = 33; 63.5%), with a median age of 25.0 years (IQR: 11.8–36.8). The overall sample of 52 patients with an acute RTI included patients presenting with an ear, sinus or throat infection (*n* = 39), a documented RTI diagnosis (*n* = 38) and with an acute cough (*n* = 20).

In total, 13 distinct symptoms were recorded among the 52 patients included in the PPS, with some patients presenting with more than one symptom (Table 1). The most reported symptoms for these patients were pharyngitis/tonsilitis (*n* = 28; 53.8%) and acute cough (*n* = 20; 38.5%). Seven (13.5%) patients also had comorbidities, as shown in Table 1.

Overall, six different antibiotics were prescribed for the 52 patients presenting with acute RTIs, with one patient receiving two antibiotics. Of these, five were oral formulations and one was an intramuscular injection. The most prescribed antibiotics were amoxicillin (*n* = 19; 35.8%), azithromycin (*n* = 12; 22.6%) and penicillin VK or phenoxymethylpenicillin (*n* = 11; 20.8%). Forty (75.5%) antibiotic prescriptions were from the Access group and 13 (24.5%) from the Watch group, with none from the Reserve group. The median prescribed antibiotic treatment duration was 5 days (IQR: 3–7 days).

More details of the patient characteristics and the PPS results for patients presenting with acute RTI symptoms are presented in Table 1.

### 2.2. Assessment of Clinimetric Properties

The achievement scores, i.e., the percentage of patients that received the care measured in the indicator, and the descriptions of the numerators and denominators that were used to calculate the level of achievement for each indicator, are presented in Table 2. Further details of the 13 RTI indicators for South Africa are included in the study by Chigome et al., 2026 [15].

Of the indicators measuring positive actions (10/13), only three (3/10, 30%) achieved a score above 85%, including one indicator assessing the use of Access antibiotics. Three of these (3/10, 30%) indicators scored below 50%. Of the indicators measuring negative actions, where a score closer to zero is the desired outcome (indicators 3, 7, 12), the indicators measuring Watch antibiotic use scored 23.1% and 30%. The indicator (#12) measuring patients at low risk of bacterial RTI receiving antibiotics scored 100%, indicating high use of antibiotics in these patients. The indicator assessing the use of Access antibiotics for patients presenting with acute cough scored 55%.

The values for each clinimetric property are presented in Table 3. All 13 of the developed indicators (100%) had applicability scores ≥10% of the reviewed medical records, with 3 (23.1%) indicators being applicable to all reviewed medical records (*n* = 52; 100%). Twelve (92.3%) indicators had a measurability score of 100%, while one (7.7%) indicator had a measurability score below 75%.

Of the 13 RTI indicators developed in the first study [15] and subsequently assessed for their clinimetric properties, 12 (92.3%) met all the predefined acceptable scores for applicability and measurability. Indicator 10 did not meet the applicability and measurability scores.

### 2.3. Prescriber Interviews to Explore Factors Currently Influencing Antibiotic Prescribing at Public Sector PHC Facilities in South Africa

Two female facility managers and two female prescribers participated in the interviews. Each facility had 7–8 full-time professional nurses and 1–2 sessional doctors who attended the clinics once a week to prescribe treatments. All the facilities had a specific register for patients presenting with acute symptoms of illnesses. On average, 1150 (SD: 465.5) patients consulted per facility per week, ranging from 600 to 1700 patients per week.

All participants indicated that they were not aware of the WHO’s AWaRe classification and there were currently no ASPs or specific antibiotic-prescribing guidelines at their facilities. Staff at three of the four facilities also stated they had not received any specific training on antibiotic prescribing and AMR, while only specified nurses had attended antibiotic-related workshops at one facility. Further details about prescriber training, AMS activities and antibiotic policies are shown in Table 4.

The factors affecting antibiotic prescribing are presented in Table 5. The main factors affecting antibiotic prescribing included antibiotic shortages (*n* = 4), patient expectations (*n* = 2), fear of complications (*n* = 2), changes to prescribing protocols (*n* = 1) and high patient volumes (*n* = 1). All participants indicated that patients expect or request antibiotics for their acute RTIs. None of the prescribers indicated that their workload affected their ability to follow prescribing guidelines.

At the time of the study, there were no specific audits for antibiotic prescribing at any of the four facilities, as presented in Table 6. The participants indicated that prescription audits, e.g., the Ideal Clinic audits and other district audits, may include antibiotic prescriptions; however, these audits are not specific for antibiotic prescribing.

All facilities had at least one quality audit between April and October 2025. The audits were conducted by capturing data from paper-based patient records either manually (*n* = 1) or electronically (*n* = 3). District managers (*n* = 3) and district quality assurance teams (*n* = 2) are responsible for prescription audits at the PHC facilities. However, at the time of data collection, none of the participating facilities had used quality indicators to assess the quality of antibiotic prescribing in their facility. All participants agreed that the implementation of quality indicators to assess antibiotic prescribing would be beneficial for their facility and improve adherence to guidelines. In addition, their implementation would not negatively impact prescribing practices at the facility. The participants’ understanding of quality assessment and recommendations to improve antibiotic prescribing are also presented in Table 6.

## 3. Discussion

This study focused on pilot testing a subset of RTI quality indicators developed in our first study [15]. The PPS results showed that 100% of patients presenting with acute RTIs received antibiotics. This is a high prescribing rate in primary care settings, where RTIs are often viral and do not require antibiotics. These findings reinforce the need for implementation of RTI quality indicators in South African PHC facilities to improve the appropriateness of their antibiotic prescribing. The results of the interviews further emphasize the need for targeted AMS interventions to address factors influencing antibiotic prescribing for RTIs. This included patient expectations, fear of complications and the lack of specific training on antibiotic prescribing in public PHC facilities.

Of the 13 RTI quality indicators tested in this study, 12 (92.3%) met all the acceptable scores for measurability and applicability. This is important, as their usefulness depends not only on their clinical relevance but also on the robustness of their clinimetric properties [39,49]. All the indicators tested in this study were deemed applicable, showing that these quality indicators are relevant to the South African PHC context, further validating their appropriateness following the RAM that was previously undertaken in South Africa [15].

Clinimetric testing is critical, as many of the proposed indicators for assessing the quality of antibiotic prescribing in real-world settings have not been sufficiently evaluated with respect to their clinimetric properties [36,39,50,51]. Here, we show that these indicators are measurable using paper-based patient records, which is common among LMICs [15]. Most quality indicators used for antibiotic prescribing whose clinimetric properties have been tested are typically for high-income countries, and electronic health records and electronic databases were used to assess the performance of these indicators [24,36,50,52,53,54].

For this study, while we assessed the achievement score of each indicator based on the PPS results, we did not set target values or thresholds for individual indicators. The low achievement scores for most indicators and all RTI patients receiving antibiotics highlight the need to address factors that may affect the successful assessment of antibiotic-prescribing quality in routine clinical practice among PHC facilities across South Africa. The low scores were favorable for those indicators assessing the proportion of Watch antibiotics, which are not recommended as first line treatment for any RTI [28,29]. In practice, facilities and districts will have to set target values and performance thresholds for each indicator, in alignment with national targets and guidelines. Determining contextually appropriate benchmarks, or performance thresholds, and subsequently interpreting the performance scores may be challenging for RTI indicators. This is because some of the indicators assess inappropriate or negative prescribing practices. This includes the proportion of patients receiving Watch antibiotics for RTIs or patients receiving Access or Watch antibiotics for acute cough. Ideally, the performance score for these negative indicators should be zero or as low as possible based on the clinical indication. Consequently, setting thresholds in practice requires careful consideration to enable accurate measurement of the appropriateness of antibiotic prescribing for RTIs and to facilitate the appropriate interpretation of any audit of antibiotic-prescribing patterns. A threshold will also need to be considered for the appropriate proportion of patients with acute RTIs who are subsequently prescribed any antibiotic.

Generally, both the AWaRe guidance and the local STGs/EML recommend the use of Access antibiotics and are aligned for treatment durations with RTIs [28,29,55], as shown in Supplementary Table S1. However, the local guidelines recommend prescribing Watch antibiotics, such as azithromycin, when there is a penicillin allergy [55]. While most antibiotics prescribed in the PPS were Access antibiotics, the use of azithromycin was high (22.6%), with no clear indication for its use. Consequently, documenting the patient’s allergy status is essential for determining whether azithromycin is clinically indicated and appropriately prescribed. This is important because the interviewed HCPs indicated they adhere to the national STGs when prescribing; however, some of the prescriptions from the PPS provided contrary evidence. The high burden of HIV and tuberculosis in South Africa also complicates the clinical management of RTIs among PHC facilities, which may result in the use of Watch antibiotics and longer treatment duration [56]. The interviewed participants also indicated that they sometimes prescribed antibiotics due to fear of complications, particularly for immunocompromised patients who may require broader spectrum antibiotics.

Whilst the indicators were measurable using paper-based records, their consistent implementation and scalability may be a challenge in the absence of electronic health records. The indicators that were difficult to measure from paper records, because they required information such as documented RTI, allergy status or treatment severity, may be improved with the use of electronic records. Interview participants recommended the use of electronic prescribing and clinical decision-support tools to improve prescribing in primary care. Going forward, it is critical for LMICs to address concerns regarding available information technology systems alongside unreliable and incomplete data in health care, especially in primary care [57], to improve future antibiotic use. As a result, this will reduce the growing burden of AMR across Africa, including South Africa [1,6,20,58,59]. Without such developments, the interoperability of health information recording and data sharing within countries remain challenging, especially among LMICs [59,60]. Electronic health information systems will not only help improve data quality, and the feasibility of the indicators, but they have also shown significant potential to improve antibiotic selection and reduce inappropriate prescribing [59,61,62]. Electronic tools may also prompt prescribers to include all vital information in patients’ records, e.g., their allergy status to potentially prescribed antibiotics. Strengthening digital integration will be advocated as part of the national action plan to improve the government’s efforts to reduce the current high burden of AMR in South Africa.

Health system challenges such as antibiotic availability and supply chain constraints complicate the measurement and interpretation of AWaRe indicators in practice among LMICs [50,63,64,65]. From the interview responses, prescribing practices may be influenced by antibiotic availability within ambulatory care public sector facilities and therefore potentially affect prescribing quality. Shortages of first-line or Access antibiotics within the PHC sector may compel prescribers to use alternative antibiotics classified within the Watch group. However, this situation may not be universal across South Africa, with a recent study among private sector pharmacies by Maluleke et al. (2025) documenting considerable prescribing of antibiotics for patients with URTIs with little dispensing of antibiotics without a prescription for this indication [66]. Where antibiotics were dispensed without a prescription, this was principally for patients with sexually transmitted infections (STIs) [66]. This mirrored a previous study in South Africa where there was little dispensing of antibiotics without a prescription for simulated patients with URTIs [67]. Indicators that consider the proportions of different AWaRe categories should be interpreted alongside contextual information on antibiotic availability. Strengthening antibiotic supply chains among public sector facilities in all provinces, where necessary, should be an AMS priority to help optimize future antibiotic use. Lack of diagnostic capacity among ambulatory care public sector facilities is another health system challenge that compromises the validity of the indicators in practice, as antibiotic prescribing in South African public sector clinics is largely syndromic [13,15,68,69]. Indicators that assume diagnostic certainty or require a diagnosis to determine prescribing appropriateness, particularly for viral RTIs, may be difficult to measure in practice, as shown in this study. When interpreting such indicators, this limitation should be considered. In addition, substantial variation in antibiotic-prescribing practices, poor adherence to treatment guidelines and high antibiotic use have been seen among PHC facilities across South Africa, highlighting the need for multifaceted ASPs and point-of-care testing in primary care to optimize future antibiotic prescribing [11,12,13,14,15,68,69].

The prescriber interviews identified other key issues that need to be addressed for the successful implementation of the quality indicators. These include current poor awareness of the AWaRe classification, the lack of prescriber training on AMS and the lack of ASPs at PHC facilities. In South African PHC facilities, antibiotic prescribing and other health services are primarily offered by nurses [15,63,64,70]. Consequently, targeted training for all HCPs in primary care, particularly nurses, to promote awareness and use of the WHO’s AWaRe classification and AMS principles, documentation practices and quality indicators could be implemented to improve future prescribing quality, data quality and acceptance of the indicators [60,65]. AMS activities that foster continuing professional development among prescribers in primary care should now be integrated into broader national policies including the South African AMR National Strategy Framework [21,71].

The quality indicators can subsequently be incorporated into the Ideal Clinic audits and other quality improvement initiatives to improve future antibiotic prescribing in South African public PHC facilities [17,72]. Key quality indicators can also subsequently be implemented using a phased approach. This list of indicators will need to be tested in a wider population of South African public PHC facilities, alongside discussions surrounding appropriate thresholds, before finalizing a set of indicators to implement. It is important that the AWaRe categories and guidance considered by any quality indicator should fully reflect local clinical guidelines, resistance patterns and other context-specific factors [28,29,34]. This refinement process will be further explored and strengthened in subsequent studies.

These indicators can also subsequently be used for specific ASPs aimed at improving the quality of antibiotic prescribing for high-priority infections of concern. Training prescribers, pharmacists and health information staff on AMS principles, documentation standards and data interpretation can also improve both data quality and the acceptance of any implemented indicator [15].

We believe a key strength of this study is the integration of the findings from the PPS with prescriber interviews to evaluate the clinimetric properties of potential RTI quality indicators. Subsequently, we identified factors that may constrain the implementation of these indicators in South African ambulatory care public sector facilities. South Africa is the first LMIC where this activity has been undertaken with quality indicators nationally adapted using the RAM by a national multidisciplinary expert panel [15]. The indicators were adapted from global model indicators developed primarily for LMICs by a global panel, which took into consideration both national and global contexts [27]. These collaborative activities demonstrate that we tested a robust set of indicators appropriate for the South African context [15]. Data for the PPS were collected from paper-based records in a standardised manner, using a previously piloted data collection instrument [73]. The pilot study focused on testing a subset of quality indicators. While the results are not generalizable to other common infection categories among ambulatory care public sector settings, they demonstrate that focusing on a subset of priority indicators is achievable in practice for paper-based periodic quality assessments whilst electronic systems are being considered. This is important considering the workload and human-resource constraints currently in PHC facilities nationally.

We acknowledge certain limitations in our study. First, we tested only a subset of the quality indicators and restricted this initial testing to a single province, as this phase was designed as a proof-of-concept as part of a feasibility study [74]. Despite this limitation, we believe vital information was obtained for each indicator. Second, we included only 52 patients presenting with acute RTI symptoms in the PPS, and only these patients were included in testing the quality indicators’ clinimetric properties. However, we believe a sample size of 52 patients is sufficient for basic analysis of clinimetric properties [75,76]. Third, data were collected over a short period and the results may not account for seasonal variations; however, this mainly affects the achievement scores. Last, data were retrospectively collected after each consultation, which means that some relevant information could not be identified, e.g., a patient’s allergy status or when only symptoms were listed and there was no specific RTI diagnosis.

Future research activities will include building on these findings and extending the testing of the clinimetric properties of quality indicators for other common infection categories where paper-based records remain the primary source of clinical data. In addition, activities to promote AWaRe-based educational interventions for prescribers in primary care, focusing on RTIs and the importance of optimizing antibiotic use, are needed given current AMR concerns across Africa and wider [1,6,65,71].

## 4. Materials and Methods

### 4.1. Study Design

The research was an exploratory feasibility study using a mixed-methods approach, which included a PPS combined with prescriber interviews conducted in November 2025 [74]. It was conducted among a convenience sample of four PHC facilities in Gauteng province. These four PHC facilities had previously taken part in the Antibiotic Prescribing in Primary Healthcare Point Prevalence Survey (APC-PPS) study, which is part of the ‘Antibiotic Data to Inform Local Action’ (ADILA) project [73]. The aim of the ADILA project was to obtain a better understanding of current presentation rates of clinical infections and antibiotic-prescribing patterns for identified infections among PHC facilities across countries [73]. The purpose of the PPS was to retrospectively pilot test the clinimetric properties, such as measurability and applicability, for 13 quality indicators for RTIs.

Interviews were conducted to identify factors that influence antibiotic prescribing in the South African primary care public sector context; assess the prescriber’s awareness of AMR, the WHO AWaRe system and quality indicators; identify current AMS initiatives in primary care; identify training gaps in relation to antibiotic prescribing and stewardship; determine the potential acceptability of using quality indicators in practice; and gain perspectives on the factors that may potentially affect the implementation of quality indicators for antibiotic prescribing in public sector settings. Obtaining this kind of information is important given concerns with current antibiotic prescribing habits among healthcare professionals operating in primary care settings across South Africa [11,12,13,14,16,20,66,67,68,69].

### 4.2. Point Prevalence Surveys

Two PPSs were conducted at each facility over a two-week period. Each survey was conducted for half a business day (approximately 4 h), representative of a standard clinic session. Data were retrospectively collected. All data entries by the data collector were verified by AKC before leaving the facility.

All children and adults presenting with acute infection symptoms (present for <14 days) on the day of the survey were included in the PPS. Patients presenting to the facilities seeking care for underlying chronic conditions as their primary reason for consultation were excluded unless they also had acute signs of infection. Patient files that did not indicate the date of onset of symptoms of acute infection were also excluded.

Basic consultation data were collected retrospectively, from patient files, for all 121 eligible patients after each patient consultation. Data were collected by the main author (AKC) and one data collector who had previously collected data in the APC-PPS study. For the 96 patients who received at least one antibiotic, in-depth consultation data were collected using a previously validated and piloted data collection instrument (Supplementary Table S2) [73]. Data collected included demographics, underlying conditions, presenting infection symptoms and prescribed antibiotics. All data were collected anonymously using paper data collection forms and inputted into a structured Microsoft Excel sheet.

### 4.3. Interviews

One interview was conducted per facility, using a semi-structured questionnaire developed by the researchers using expert opinion and literature, taking into consideration the results from previously conducted PPS studies [12,15,77,78]. The questionnaire used for the interviews is available in Supplementary Table S3.

Interviews were conducted face-to-face by the main author (AKC) with the facility manager or one senior prescriber involved in the management of patients presenting with acute infection symptoms on the first day of the PPS at each of the four facilities. Interviews were conducted in a private room in the facility and were audio-recorded. All participants provided written informed consent for participation and audio-recording prior to the start of the interview.

All interview responses were manually transcribed on a Microsoft Excel™ spreadsheet. Open-ended responses from the interview were transcribed verbatim by listening to the audio recordings. A content analysis approach associated with qualitative research was used to systematically analyse the explanations from the open-ended responses. Categories were generated, aimed at capturing the commonalities and/or nuances in the responses. Responses were subsequently coded according to the categories. Direct quotes were in the presentation of the results to illustrate the identified categories. No qualitative data software packages were used for the analysis of the interview responses.

### 4.4. Data Analysis and Assessing the Clinimetric Properties of the Quality Indicators

All data from the PPSs were captured on a Microsoft Excel™ spreadsheet. After cleaning, the data were imported into jamovi^®^ (version 2.7.15) for descriptive statistical analysis. The inclusion criterion for the clinimetric assessment was subsequently narrowed from the 121 eligible patients from the PPSs to just 52 patients presenting with at least one acute RTI-related symptom. Patients not presenting with at least one acute RTI-related symptom were not included in the clinimetric assessment. The inclusion criteria for the PPS and the clinimetric assessment are presented in Supplementary Figure S1.

The clinimetric properties of these 52 patients were tested using the pre-determined acceptable ranges and definitions shown in Table 7. For each of the 13 indicators, numerators and denominators were defined to quantify the achievement score as a percentage. The numerator was defined as the population who received the recommended care or patients with the recommended outcome [24,39]. The denominator was defined as the target population who should receive the recommended care or the total number of eligible patients [24,39]. For indicators 1 to 3, the denominator in this study considered all patients presenting with any acute RTI-related symptom. However, to enable assessment, the applicability of the denominator for an indicator reflected the recorded symptoms. For indicator 4, the denominator considered patients presenting with acute ear, sinus or throat infection symptoms only. For indicators 5 and 6, the denominator considered patients presenting with acute ear, sinus or throat infection symptoms at high risk of severe complications. For indicator 7, the denominator considered patients with any acute RTI symptom at low risk of severe complications. For indicators 8 and 9, the denominator considered patients with a documented specific RTI diagnosis, e.g., acute sinusitis not listed symptoms such as acute cough or chest pain, to assess alignment with the WHO AWaRe guidelines. For indicators 10 to 13, the denominator considered patients with an acute cough. The varied denominator allowed for sensitivity of analysis of applicability for discrete sub-sets of patients within the overall denominator as applied to indicators 1 to 3.

Where applicable, an RTI diagnosis was defined according to a documented bacterial infection diagnosis in the WHO AWaRe antibiotic book and national treatment guidelines, as shown in Supplementary Table S1 [28,29,46]. In addition, where applicable, a high-risk patient was defined according to the criteria described in the WHO AWaRe antibiotic book, considering the documented bacterial infection diagnosis; the severity of symptoms, e.g., fever; the risk of complications due to comorbidities, including immunosuppression, patients living with HIV or patients with lung/heart/liver/kidney disease; and the risk of rheumatic fever for pharyngitis, and bilateral otitis in children below two years for otitis media [28,29].

Assessment of the clinimetric properties of the indicators, i.e., their applicability, measurability and achievement was performed using Microsoft Excel™.

## 5. Conclusions

In conclusion, this exploratory study evaluated the clinimetric properties of 13 RTI indicators and highlighted important contextual factors that may affect the clinimetric performance of these quality indicators in public sector PHC facilities in South Africa. Overall, the indicators demonstrated acceptable performance for achievement, applicability and measurability. Interviews with primary care prescribers identified the need for educational and regulatory activities to be implemented alongside quality indicators to promote the use of the WHO’s AWaRe classification and adapted clinical guidance.

The next steps involve further research on the identified indicators in practice among PHCs in South Africa and across LMICs, building on this feasibility study.

## Figures and Tables

**Table 1:** Study sample characteristics and summary of PPS results (*n* = 52).

Characteristic		Number of Patients (%, *N* = 52)
No. of patients with acute RTI symptoms per facility	Facility A	10 (19.2%)
	Facility B	12 (23.1%)
	Facility C	13 (25.0%)
	Facility D	17 (32.7%)
Distribution of patients, by sex	Female	33 (63.5%)
	Male	19 (36.5%)
Comorbidities (7 patients total, 13%) *	Diabetes	4 (7.7%)
	Hypertension	4 (7.7%)
	HIV	3 (5.8%)
	COPD	2 (3.8%)
	Asthma	1 (1.9%)
Fever reported	Yes	15 (28.8%)
	No	37 (71.2%)
Acute infection symptoms **	Tonsilitis/pharyngitis/sore throat	28 (53.8%)
	Acute cough	20 (38.5%)
	Acute otitis media/ear pain	14 (26.9%)
	Nasal congestion	8 (15.4%)
	Chest pain	7 (13.5%)
	Headache	5 (9.4%)
	Runny nose	5 (9.4%)
	Runny nose with yellow/green discharge	5 (9.4%)
	Bronchitis	4 (7.4%)
	Bacterial sinusitis	3 (5.7%)
	Shortness of breath	2 (3.8%)
	Bacterial rhinitis	1 (1.9%)
	Sinusitis	1 (1.9%)
Prescribed antibiotics (n = 53) ***	Amoxicillin (Access group)	19 (35.8%)
	Azithromycin (Watch group)	12 (22.6%)
	Penicillin VK (Access group)	11 (20.8%)
	Amoxicillin/clavulanic acid (Access group)	5 (9.4%)
	Cephalexin (Access group)	5 (9.4%)
	Ceftriaxone (Watch group)	1 (1.9%)
Treatment duration (days) (median, IQR)	5 days (IQR: 3–7)	
Treatment duration (days) ****	5	23 (43.4%)
	7	14 (26.4%)
	3	11 (20.8%)
	10	3 (5.7%)
	6	1 (1.9%)
	Stat dose	1 (1.9%)

COPD = chronic obstructive pulmonary disease; HIV = human immunodeficiency virus; Q1 = First quartile; Q3 = Third quartile; SD = standard deviation; * some patients had more than one comorbid condition; ** some patients had more than one symptom; *** one patient received more than one antibiotic; **** represents individual antibiotic courses rather than the number of patients as some patients received more than one antibiotic; Access and Watch—AWaRe classification [42].

**Table 2:** Achievement scores for quality indicators.

No.	Indicator	AWaRe Category Addressed by Indicator	Numerator Description (*n*)	Denominator Description (*N*)	Achievement	
					*n*/*N*	%
1.	Proportion of all patients presenting with an acute RTI given oral amoxicillin	Access	No. of patients with an acute RTI given oral amoxicillin	No. of patients with an acute RTI	19/52	36.5
2.	Proportion of all patients presenting with an acute RTI given an oral Access antibiotic, including amoxicillin	Access	No. of patients with an acute RTI given an oral Access antibiotic, including amoxicillin		40/52	76.9
3.	Proportion of all patients presenting with an acute RTI given an oral Watch antibiotic **	Watch	No. of patients with an acute RTI given an oral Watch antibiotic		12/52	23.1
4.	Proportion of patients with an ear/sinus/throat infection (not pneumonia) given an oral antibiotic	Access, Watch	No. of patients with an ear/sinus/throat infection (not pneumonia) given an oral antibiotic	No. of patients with an ear/sinus/throat infection	39/39 ^a^	100
5.	Proportion of patients with an ear/sinus/throat infection (not pneumonia) at high risk * of severe complications given amoxicillin	Access	No. of patients with an ear/sinus/throat infection at high risk of severe complications given amoxicillin	No. of patients with an ear/sinus/throat infection at high risk of severe complications	6/16	37.5
6.	Proportion of patients with an ear/sinus/throat infection (not pneumonia) at high risk * of severe complications given an oral Access antibiotic (including amoxicillin)	Access	No. of patients with an ear/sinus/throat infection at high risk of severe complications given an oral Access antibiotic (including amoxicillin)		15/16	93.8
7.	Proportion of patients at lower risk * of a bacterial RTI given an oral antibiotic **	Access, Watch	No. of patients at lower risk of bacterial RTI given an oral antibiotic	No. of patients at lower risk * of a bacterial RTI	33/33	100
8.	Proportion of patients with a documented RTI diagnosis given the duration in days of oral antibiotics recommended in the WHO AWaRe Antibiotic Book	Access, Watch	No. of patients with a documented RTI diagnosis given the duration in days of oral antibiotics recommended in the WHO AWaRe Antibiotic Book	No. of patients with a documented RTI diagnosis given an antibiotic	15/38 ^b^	39.5
9.	Proportion of patients with a documented bacterial RTI diagnosis given an oral Access or Watch antibiotic	Access, Watch	No. of patients with a documented bacterial RTI diagnosis given an oral Access or Watch antibiotic	No. of patients with documented acute bacterial RTIs	38/38 ^b^	100
10.	Proportion of patients (no relevant comorbidities) presenting with acute cough given an antibiotic who met WHO AWaRe guidelines for antibiotic prescription ***	Access, Watch	No. of patients with no relevant comorbidities presenting with acute cough who met AWaRe guidelines for antibiotic prescription	No. of patients (no relevant comorbidities) with acute cough	8/20 ^c^	40
11.	Proportion of patients (no relevant comorbidities) presenting with acute cough prescribed an Access antibiotic	Access	No. of patients with no relevant comorbidities presenting with acute cough prescribed an Access antibiotic		11/20 ^c^	55
12.	Proportion of patients (no relevant comorbidities) presenting with acute cough prescribed a Watch antibiotic **	Watch	No. of patients with no relevant comorbidities presenting with acute cough prescribed a Watch antibiotic	No. of patients with acute cough	6/20 ^c^	30
13.	Proportion of acute cough cases given an antibiotic with documented bacterial indications (clinical justification for antibiotic use where the documented signs are suggestive of bacterial infection e.g., fever > 38 °C, purulent sputum, dyspnea, or suspected pneumonia)	Access, Watch	No. of acute cough cases with documented bacterial indications	No. of acute cough cases given an antibiotic	12/20 ^c^	60

The 13 indicators for RTIs were developed in the first study by Chigome et al., 2026 [15]. * Considered the documented bacterial infection diagnosis; the severity of symptoms e.g., fever; and the risk of complications due to comorbidities, including immunosuppression, patients living with HIV or patients with lung/heart/liver/kidney disease. Considered the risk of rheumatic fever for pharyngitis, bilateral otitis in children below 2 years for otitis media, as described in the WHO AWaRe antibiotic book [29,43]; ** These indicators are considered negative indicators, where Watch antibiotics and antibiotics for low-risk patients are not recommended in guidelines (see Table S1) and lower achievement scores are better. *** Patients with acute cough associated with acute RTIs (suspected/confirmed) [29,43]. ^a^ The denominator is 39, as only patients with an ear, sinus or throat infection were included. ^b^ The denominator is 38, as only patients with a documented RTI diagnosis were assessed for alignment of duration with the WHO AWaRe guidelines for the respective RTI. ^c^ The denominator is 20, as only patients presenting with acute cough were included.

**Table 3:** Assessment of applicability and feasibility of the quality indicators.

No.	Indicator	Applicability		Measurability	
		*n*/*N* ^1^	%	*n*/*N* ^1^	%
1.	Proportion of all patients presenting with an acute RTI given oral amoxicillin	52/52	100	52/52	100
2.	Proportion of all patients presenting with an acute RTI given an oral Access antibiotic, including amoxicillin	52/52	100	52/52	100
3.	Proportion of all patients presenting with an acute RTI given an oral Watch antibiotic	52/52	100	52/52	100
4.	Proportion of patients with an ear/sinus/throat infection (not pneumonia) given an oral antibiotic	39/52	75	39/39	100
5.	Proportion of patients with an ear/sinus/throat infection (not pneumonia) at high risk * of severe complications given amoxicillin	16/52	30.8	16/16	100
6.	Proportion of patients with an ear/sinus/throat infection (not pneumonia) at high risk * of severe complications given an oral Access antibiotic (including amoxicillin)	16/52	30.8	16/16	100
7.	Proportion of patients at lower risk ** of a bacterial RTI given an oral antibiotic	33/52	63.5	33/33	100
8.	Proportion of patients with a documented RTI diagnosis given the duration in days of oral antibiotics recommended in the WHO AWaRe Antibiotic Book	38/52	73.1	38/38	100
9.	Proportion of patients with bacterial RTIs given oral Access or Watch antibiotics	38/52	73.1	38/38	100
10.	Proportion of patients (no relevant comorbidities) presenting with acute cough who met WHO AWaRe guidelines for antibiotic prescription **	20/52	38.5	10/20	50
11.	Proportion of patients (no relevant comorbidities) presenting with acute cough prescribed Access antibiotics	20/52	38.5	20/20	100
12.	Proportion of patients (no relevant comorbidities) presenting with acute cough prescribed Watch antibiotics	20/52	38.5	20/20	100
13.	Proportion of acute cough cases with documented bacterial indications (clinical justification for antibiotic use where the documented signs are suggestive of bacterial infection e.g., fever > 38 °C, purulent sputum, dyspnea, or suspected pneumonia)	20/52	38.5	20/20	100

The 13 indicators for RTIs were developed in the first study by Chigome et al., 2026 [15]. ^1^ = n represents the numerator for each indicator and N represents the denominator for each indicator; * Considered the documented bacterial infection diagnosis; the severity of symptoms e.g., fever; and the risk of complications due to comorbidities, including immunosuppression, patients living with human immunodeficiency virus (HIV) or patients with lung/heart/liver/kidney disease. In addition, considered the risk of rheumatic fever for pharyngitis, bilateral otitis in children below 2 years for otitis media, as described in the WHO AWaRe antibiotic book [29,43]; ** Patients with acute cough associated with acute RTIs (suspected/confirmed); AWaRe = Access, Watch, Reserve [8,29]; RTIs = respiratory tract infections; WHO = World Health Organization.

**Table 4:** Prescriber training, antimicrobial stewardship and antibiotic-prescribing policies.

Interview Question	Response	Number (*n* = 4)	Verbatim Comments (Quotes) from Participants
Have your staff received specific training on antibiotic prescribing and AMR?	Yes	1	*“Specific staff have been nominated to attend workshops at the facility; not all staff have received training.”* *“No, the district office identifies specific personnel to attend specific workshops based on an identified need.”*
	No	3	
Does your facility have specific antibiotic-prescribing protocols or policies?	Yes	0	*“No, we use the STGs and EML.”* *“We use the APC and IMCI guidelines together with the EML for prescribing.”*
	No	4	
Do you use the South African STGs and EML for PHC Level?	Yes	4	Not applicable
	No	0	
Which other guidelines influence antibiotic prescribing at your facility?	APC	3	*“We use the STI guidelines and the APC guidelines for acute cough.”* *“We have our guidelines on display for referral to assist us when consulting patients with infections and other health conditions.”*
	IMCI	3	
	STI	1	
Do you know about the WHO’s AWaRe antibiotic classification? If yes, does your facility consider the AWaRe guidance?	Yes	0	*“No, I know about the World Health Organization, but I have never heard of the classification.”*
	No	4	

AMR = antimicrobial resistance; APC = Adult Primary Care clinical tool [44]; AWaRe = Access, Watch, Reserve [8]; IMCI = Integrated Management of Childhood Illness guidelines [45]; PHC = primary healthcare; STGs and EML = Standard Treatment Guidelines and Essential Medicines List [46]; STI guidelines = Sexually Transmitted Infection Management Guidelines [47]; WHO = World Health Organization.

**Table 5:** Factors affecting antibiotic prescribing.

Interview Question	Response	Number (*n* = 4)	Verbatim Comments (Quotes) from Participants *
How do you explain to patients when antibiotics are not needed?	Explain that symptoms are viral and self-limiting and an antibiotic is not needed	4	*“Explain that they don’t need an antibiotic based on their symptoms and tell them about resistance to antibiotics.”* *“Antibiotics are prescribed according to need not request and they tell them about AMR.”*
	Tell them about AMR	2	
	Suggest home remedies	1	
Does your workload affect your ability to follow prescribing guidelines?	Yes	0	*“No, we have to refer some patients due to excessive workload, but we still follow the guidelines.”* *“A high influx of patients affects stock availability so we may end up prescribing alternative antibiotics, but we still follow our guidelines.”*
	No	4	
What factors influence antibiotic prescribing at your facility?	IMCI guidelines	2	*“We consider guidelines on symptoms in the IMCI guideline* e.g., *fever, productive cough or if the patient is consulting for the second time for the same symptoms.”* *“We use guidelines, especially IMCI guidelines to check if it is a self-limiting infection before prescribing. We consider complications for immunocompromised patients and malnourished children.”* *“We check the presence of infections, or if we suspect an infection based on symptoms. Sometimes a patient will request an antibiotic.”*
	Patient expectations	2	
	The presence of infection	1	
	Fear of complications	2	
	Patient consulting for the second time for the same symptoms	1	
What challenges do you face when prescribing antibiotics at your facility?	Stock outs/supply chain constraints	4	*“We have the EML and SVS, so we monitor stock availability weekly and place emergency orders if required. We give alternate treatment recommended in the EML, so shortages don’t really affect us.”* *“Manual patient records affect history taking as patients sometimes consult at more than one facility. Without proper history taking, sometimes one may not give the right antibiotic and may not ask the patient the right questions. Language barriers affect consultation and making the right diagnosis.”*
	Changes to prescribing protocols	1	
	High patient volumes	1	
	Manual patient records	1	
	Language barriers	1	
Describe how the challenges influence the choice of antibiotics prescribed.	Dispense alternative treatment	3	*“We identify substitutes if the district tells us an antibiotic is out of stock.”* *“We use our discretion on the next best alternative, taking into consideration patient allergies.”*
	Place emergency orders	1	
Do antibiotic shortages affect your prescribing decisions?	Yes	3	*“The facility places emergency orders.”*
	No	1	
What support or resources would help you improve antibiotic prescribing?	Training	3	*“Stock availability and more staff.”* *“Training on the AWaRe system you mentioned.”*
	Additional human resources	1	
	An AMS committee at PHC level	1	
	Improved stock availability	1	

AMS = antimicrobial stewardship; AMR = antimicrobial resistance; EML = Essential Medicines List [46]; IMCI = Integrated Management of Childhood Illness [45]; PHC = primary healthcare; SVS = Stock Visibility System [48]; * The comments are direct quotes from the interview participants.

**Table 6:** Quality assessment of antibiotic prescribing (*n* = 4).

Interview Question	Response	Number (n = 4)	Verbatim Comments (Quotes) from Participants *
Is antibiotic prescribing reviewed or audited at your facility?	There are no specific audits for antibiotic prescribing. Prescription audits. e.g., the Ideal Clinic audits and the district office audits, include antibiotic prescriptions but are not specific for antibiotic prescribing	4	*“No specific antibiotic audit. General file audit is conducted quarterly by district managers and IDEAL clinic audit once a year, National Core Standards audit is every 5 years.”* *“Not specific for antibiotic prescribing. Pharmacy audits are conducted. Regional audits are also conducted by the quality assurance team (EUREKA) where the whole file is audited.”* *“Not specific for antibiotics but we have IDEAL clinic quality audits once a year and the district office audits files once a year.”* *“Prescription audits are not specific for antibiotic prescribing,* e.g., *the IDEAL clinic audits.”*
Have you received feedback on the audit? If yes, please describe the outcome of the audit and what activities are generally undertaken following the dissemination of the findings?	Yes, the facility manager receives feedback and a checklist to guide the implementation of appropriate QIPs and recommended training	4	*“Yes, facility manager receives feedback and is required to develop appropriate quality improvement projects (QIPs) based on audit findings. The district follows up in 6 months.”*
What is your understanding of quality indicators?	Prescribing right the first time, doing it on time, for the right patient using the right documents	1	Not applicable
	Have never heard about them, facility uses performance indicators	1	
	Assessing if you are adhering to guidelines and if you are giving quality care to the patient	1	
	They are used to meet performance targets, e.g., make sure all children are fully immunized and prescribe according to the EML	1	
Please explain why you have not used quality indicators to assess the quality of antibiotic prescribing at your facility?	Not a requirement/standard procedure	3	*“We don’t have the indicators. Time may also be the reason.”* *“Not protocol, PHC managers check prescriptions but not specifically antibiotic prescriptions.”* *“Not part of requirements from the district, workload may also not allow.”*
	Unavailability of indicators	1	
	Time and resource constraints	2	
Recommendations by participants	Recommend electronic prescribing and electronic clinical decision support tools *	3	*“The facility has some computers, but we no longer have the system that used to help us with patient consultations. We would appreciate having an electronic system to assist with prescribing at the facility.”* *“Electronic prescribing will be beneficial to us especially for new staff, to provide quicker service, avoid mistakes, avoid duplication of therapy and because manual files get lost.”* *“Training to empower employees and moving from paper-based prescribing to electronic prescribing.”*
	Recommend training to equip employees	1	
	No recommendations	1	

EML = Essential Medicines List; PHC = primary healthcare; QIP = quality improvement project; * The comments are direct quotes from the interview participants.

**Table 7:** Attributes and clinimetric properties of clinical indicators [24,36,39,78,79].

Attribute/Property	Definition
**Applicability**	The proportion of all medical records for which the indicator could be applied or the percentage of patients to whom the indicator applies. Generally, a quality indicator should be applicable to ≥10% of the reviewed medical records (with a minimum of 10 patients per facility). Overall, a quality indicator can be considered applicable if it can be calculated from data extracted from ≥75% of the medical records in facilities.
**Feasibility**	Data are available in current PHC practice systems i.e., electronic and/or paper-based medical records.
**Measurability**	Data required to calculate the numerator and denominator of an indicator are readily available and can be collected with sufficient accuracy and consistency from patient medical records. Generally, an indicator is considered measurable if data are available for ≥75% of medical records and data are missing in <25% of cases.
**Validity** **(appropriateness)**	The extent to which an indicator is beneficial, effective and evidence-based (or clinically indicated) when applied in primary care.

## Data Availability

The original contributions presented in this study are included in the article/Supplementary Materials. Further reasonable inquiries can be directed to the corresponding authors for consideration.

## Supplementary Materials

The following supporting information can be downloaded at: https://www.mdpi.com/article/10.3390/antibiotics15060562/s1, Supplementary Table S1 provides a summary of treatment guidance for acute RTIs from the WHO AWaRe antibiotic book and the South African treatment guidelines [8,28,29,44,45,46]. Supplementary Table S2 contains the data collection sheet used for the PPS [73]. Supplementary Table S3 contains the semi-structured interview questionnaire used for the prescriber interviews. Supplementary Figure S1 contains the inclusion and exclusion criteria for the PPS and clinimetric assessment [29,43].
