# Supplementary material for: The Applicability of AWaRe-Based Antibiotic Quality Indicators to Assess the Appropriateness of Antibiotic Prescribing in Primary Healthcare in South Africa: A Multicentre Point Prevalence Study and Implications for the Future
Source: Antibiotics (Basel). 2026 Jun 1;15(6):562. doi: 10.3390/antibiotics15060562 (PMC13296314; doi:10.3390/antibiotics15060562)
Supplement: Supplementary file 1 [file antibiotics-15-00562-s001.zip › antibiotics-4256223-supplementary.pdf]

Supplementary Tables

Supplementary Table S1: Summary of guidance for acute RTIs from the WHO AWaRe antibiotic book and the South African treatment guidelines (Adapted from 8,28,29,44-46).

Infection symptoms/diagnosis	AWaRe Antibiotic Book	STGs/EML Primary care	APC adult guidelines	IMCI pediatric guidelines
**Bronchitis**	• Usually presents as a persistent cough which may take weeks to resolve.<br>• Almost all cases are viral and self-limiting; patients should be informed that cough can last several weeks.<br>• Yellow/green colour of the sputum does not indicate bacterial infection and the need for antibiotics.<br>• Antibiotic treatment is not recommended and should be avoided as there is no evidence of a significant clinical benefit.	Antibiotics are not indicated in acute bronchitis in the absence of underlying COPD.	Antibiotics are not indicated in acute bronchitis.	Antibiotics are not indicated in acute bronchitis.
**Acute otitis media/ear pain**	• Antibiotics are not needed for most cases.<br>• Antibiotic treatment could be considered in adults and children if the symptoms are severe e.g. systemically very unwell, ear pain despite analgesics, fever ≥39.0°C.<br>• Antibiotics should also be considered for immunocompromised children or for bilateral acute otitis media in children <2 years.	• For otitis media, amoxicillin or amoxicillin/clavulanic acid are recommended orally for 5 days.<br>• For adults give amoxicillin 1500mg every 12 hours orally. The treatment duration can be extended to 10 days for	• Otitis media is suspected if a patient has a painful ear for more than 2 days or pain that wakes the patient at night or a temperature ≥ 38°C in past 2 days.<br>• Refer the patient if there is no response to treatment	• Acute ear infection is suspected if there is ear pain which wakes the child at night or pus is seen draining from the ear and discharge is reported for less than 14 days.<br>• Give ceftriaxone stat and refer urgently if mastoiditis is

 https://doi.org/10.3390/antibiotics15060562

	• First choice treatment for adults is amoxicillin 500mg every 8 hours orally for 5 days<br>• Second choice treatment for adults is amoxicillin/clavulanic acid 500/125mg every 8 hours orally for 5 days.<br>• Amoxicillin or amoxicillin/clavulanic acid orally for 5 days are the recommended treatment options for children.	children if pain or discharge persists.<br>• Amoxicillin/clavulanic acid (875/125mg twice daily for 5-10 days) should be given to patients who have taken amoxicillin in the previous 30 days or had poor response to a 10-day course of amoxicillin.	after 3 days or if mastoiditis is suspected.	suspected (if there is tender swelling behind the ear).
**Sore throat/ pharyngitis/tonsilitis**	• Antibiotics are not needed for most cases since most cases are self-limiting and of viral origin.<br>• Cases caused by Streptococcus pyogenes (group A Streptococcus) can very rarely be complicated by, for example, rheumatic fever, rheumatic heart disease and acute glomerulonephritis.<br>• The only clear indication for antibiotic treatment in adults and children is to reduce the probability of developing rheumatic fever in endemic settings (however, after 21 years of age the risk of rheumatic fever is lower).<br>• Bacterial pharyngitis has a more severe presentation that viral pharyngitis and includes fever (≥38.0 °C), tender cervical lymph nodes and pharyngeal exudates.<br>• First choice treatment is amoxicillin 500mg every 8 hours orally or phenoxymethylpenicillin (Pen VK) 500mg every 6 hours orally. The treatment duration for adults and children is 5 days if at	• Antibiotics are not required for all patients with a sore throat.<br>• Antibiotics to eradicate streptococci must be given to patients presenting with a sore throat who are at risk for rheumatic fever (3–21 years of age) if they have enlarged tonsils PLUS at least one of the following criteria:<br>- Exudates on their tonsils<br>- No cough<br>- No runny nose<br>• The recommended treatment is benzathine benzylpenicillin single dose OR oral amoxicillin for 10 days for children	• Suspect bacterial pharyngitis/tonsilitis if patient has a sore/red throat and the patient has either enlarged tonsils with pus/white patches on tonsils OR enlarged tonsils without cough or runny nose. If not suspect viral pharyngitis and give no antibiotics.<br>• If the described symptoms are present, give recommended treatment if ≤ 21 years old. If > 21 years old, advise to return if symptoms persist/worsen.	• Suspect possible streptococcal infection if the child has a sore throat, enlarged tonsils with white or yellow exudate AND one or more of the following:<br>○ No runny nose<br>○ No cough<br>○ Scarlatiniform rash.<br>• Give benzathine benzylpenicillin IM as a single dose.<br>• Give azithromycin if the child is allergic to penicillin.<br>• Only give oral phenoxymethylpenicillin if the caregiver does not want the child to have an injection.

	low risk of rheumatic fever and 10 days if at high risk of rheumatic fever for first line treatment.<br>• Second choice treatment is cephalexin 500mg every 8 hours orally or clarithromycin 500mg every 12 hours orally. The treatment duration is always 5 days.<br>• The same treatment is recommended for children.	and amoxicillin1000mg twice daily for 10 days for adults OR oral phenoxymethylpenicillin 500mg twice daily for 10 days.<br>• Azithromycin is recommended for penicillin allergy (500mg daily for 3 days).		
**Acute sinusitis**	• Most cases of sinusitis occur as a complication of a viral upper respiratory tract infection and are self-limited.<br>• Yellow/green coloured nasal discharge alone is not a sign of bacterial infection and not an indication for antibiotic treatment.<br>• If antibiotic treatment is required, amoxicillin has good activity against Streptococcus pneumoniae, the most common bacterial cause of acute bacterial sinusitis.<br>• Acute bacterial sinusitis should be suspected when signs/symptoms persist ≥10 days without improvement OR significant worsening of symptoms after initial mild phase.<br>• Antibiotics should be considered if:<br>○ Severe onset of symptoms: fever ≥39.0 °C and purulent nasal discharge or facial pain for at least 3-4 consecutive days	Children above 3 years and adults should be given amoxicillin 500mg every 8 hours for 5 days (azithromycin for penicillin allergy)	• Sinusitis should be suspected if the patient has facial pain when pushing on forehead/cheeks, headache worse on bending forward; thick nasal/postnasal discharge, recent common cold.<br>• If symptoms ≥ 10 days, fever ≥ 38°C, purulent nasal discharge, face pain ≥ 3 days, or symptoms worsen after initial improvement of common cold, give amoxicillin 500mg 8 hourly for 5 days. If severe penicillin allergy, give instead azithromycin 500mg daily for 3 days.	

	o Patients at increased risk of complications e.g. those with chronic underlying comorbid diseases (deciding on a case by-case basis)<br>o "Red flag" signs/symptoms suggestive of complicated infection such as systemic toxicity, persistent fever ≥39.0°C, periorbital redness and swelling, severe headache, or altered mental status.<br>• Recommended treatment is amoxicillin 1000mg every 8 hours or amoxicillin/clavulanic acid 500/125mg every 8 hours for 5 days.<br>• The same treatment choice is recommended for children.			
**Acute cough**	• No specific guidance on treatment, described as a symptom of acute RTIs.<br>• Describes cough with sputum production as a symptom suggestive of pneumonia (helps differentiate bronchitis from pneumonia).	No specific guidance on treatment, described as a symptom of acute RTIs, COVID-19; tuberculosis and other respiratory diseases.	For cough less than 14 days, give antibiotics (amoxicillin 1g every 8 hours for 5 days) if pneumonia is suspected. Give amoxicillin/clavulanic acid 875/125mg every 8 hours for 5 days for patients living with HIV, patients above 65 years of age, diabetic patients, alcohol misuse or patients with lung/heart/liver/kidney disease.	For cough (less than 14 days) associated with breathing difficulty, give amoxicillin for 5 days if pneumonia is suspected.

NB : AMS= antimicrobial stewardship; AMR= antimicrobial resistance; APC= Adult Primary Care guidelines for South Africa; AWaRe= Access, Watch, Reserve [8]; COPD= chronic obstructive pulmonary disease; HIV= human immunodeficiency virus; IMCI= Integrated Management of Childhood Illness guidelines for South Africa; PHC= primary healthcare; RTI= respiratory tract infection; STGs= Standard Treatment Guidelines for South African primary care; WHO= World Health Organization

antibiotics

MDPI

Supplementary Table S2: Data Collection Sheet - Consultation-level data

Facility ID code	Date of PPS (dd/mm/yyyy)	Sex Male, Female, Other	Age of the patient (0 if <1 month/neonate) Years ≥ 2 years	Age of the patient (0 if <1 month/neonate) Months 1-23 months	*Amount of time to travel to facility (minutes)*

**Does the patient have any relevant comorbidities?** *(Tick all that apply)*	O No relevant comorbidities<br>O Unknown/missing information<br>O HIV<br>O Malnutrition<br>O Diabetes	O Chronic obstructive pulmonary disease (COPD)<br>O Asthma<br>O Other chronic lung problems<br>O Chronic heart problems<br>O Other	Please specify other comorbidities:

**Has the patient already sought care / medication for this infection?**	O Yes O No O Unknown

**Does the patient have / report having a fever?**	O Yes	O No	O Unknown	If Yes,	→	O Persistent fever lasting 7 days or longer
					→	O Suspected enteric fever
					→	O Patient received anti-malarial prescription or this fever episode

**Please select all reasons the patient come to the clinic today** *(Tick all that apply)*	O Acute cough	If Yes,	→	O Cough >5 days
			→	O Shortness of breath/ difficulty breathing
			→	O Chest pain
	O Sore throat / pharyngitis/ tonsillitis			
	O Facial pain or pressure/ sinusitis			
	O Runny nose / nasal congestion / coryza			
	O Ear pain/ acute otitis media	If Yes,	→	O Uni-lateral ear pain
			→	O Bi-lateral ear pain
			→	O Otorrhoea/ ear discharge
	O Toothache/ tooth abscess			
	O Acute diarrhoea / gastroenteritis	If Yes,	→	O Bloody diarrhoea
	O Increased urgency or frequency of urination / UTI	If Yes,	→	O Blood in urine
	O Painful urination			
	O Genital discharge / STI			
	O Wound/ burn/ bite infection			
	O Skin rash / spots – without swelling			

 https://doi.org/10.3390/antibiotics15060562

	O Skin swelling / redness / warmth / pain	If Yes, →	O Swollen lymph nodes
	O Other primary presentation/ diagnosis	If Yes,	Please specify other symptoms/ diagnosis:

Please complete information regarding the antibiotic(s) prescribed:

**Antibiotic Name**	**1.**	**2.**	**3.**
**Days of antibiotic treatment prescribed**			
**Formulation of antibiotic being prescribed** *(Tablet, Capsule, Syrup, Granules, Dispersible tablet, Powder, Topical, IM, IV, Other)*	*Please specify if other formulation* ______	*Please specify if other formulation* ______	*Please specify if other formulation* ______
**Dose per unit (e.g., per tablet) of the antibiotic prescribed**			
**Unit** *(Micrograms, mg, g, IU)*			
**Number of units per day prescribed** *(e.g., number of tablets)*			
**Percent w/v of ointment/ cream**			

Supplementary annexure S3: Semi-structured interview questionnaire

Instructions: Please provide the most accurate response to the following questions regarding antibiotic prescribing at your facility.

<table>
<tr><td colspan="3">Facility Code</td><td colspan="4"></td></tr>
<tr><td colspan="3">Province</td><td colspan="4">Gauteng</td></tr>
<tr><td colspan="3">District</td><td colspan="4"></td></tr>
<tr><td colspan="3">Sub-District</td><td colspan="4"></td></tr>
<tr><td colspan="3">How many patients do you see at the facility?</td><td>Per week</td><td></td><td>Per month</td><td></td></tr>
<tr><td colspan="3">Do you have a patient facility register where all patients are recorded?</td><td colspan="2">Yes</td><td colspan="2">No</td></tr>
<tr><td colspan="3">If above answer is NO, what are you using?</td><td colspan="4"></td></tr>
<tr><td colspan="3">Do you have specific register for patients presenting with acute symptoms/illness?</td><td colspan="3">Yes</td><td>No</td></tr>
<tr><td colspan="3">If above answer is NO, how do you keep record of patients consulting for acute symptoms/illness?</td><td colspan="4"></td></tr>
<tr><td colspan="7">How many staff currently work in your clinic, in each of the following categories?</td></tr>
<tr><td>Nurses</td><td>Doctors</td><td>Pharmacists</td><td>Pharmacist assistants</td><td>Dental assistants</td><td colspan="2">Other (specify)</td></tr>
<tr><td></td><td></td><td></td><td></td><td></td><td colspan="2"></td></tr>
<tr><td colspan="3">Who is authorised to prescribe antibiotics at your facility? Please specify</td><td colspan="4"></td></tr>
<tr><td colspan="3">Have your staff received specific training on antibiotic prescribing and antimicrobial resistance?</td><td colspan="2">Yes</td><td colspan="2">No</td></tr>
<tr><td colspan="3">If above answer is YES, please specify the type of training received, who orchestrated this and when?</td><td colspan="4"></td></tr>
<tr><td colspan="3">Does your facility have specific antibiotic prescribing protocols or policies?</td><td colspan="4"></td></tr>
<tr><td colspan="3">If above answer is YES, briefly describe these protocols.</td><td colspan="4"></td></tr>
<tr><td colspan="3">Are there any antimicrobial stewardship programs (ASPs) currently being implemented at your facility especially if part of monitoring adherence to prescribing protocols?</td><td colspan="4"></td></tr>
<tr><td colspan="3">If above answer is YES, please specify the nature and type of ASPs currently being implemented.</td><td colspan="4"></td></tr>
</table>

Do you use the South African Standard Treatment Guidelines and Essential Medicine List for Primary Healthcare Level? Separately – If yes, are these included as part of antibiotic prescribing protocols being used in your facility?	Yes	No
If **NO**, please explain what you are using in the place of the Standard Treatment Guidelines and Essential Medicine List for Primary Healthcare Level.		

Which other antibiotic guidelines are available in your facility? Do they also influence antibiotic prescribing at your facility? If so – in what way?	
How often do you refer to these guidelines when prescribing antibiotics?	
Does your workload affect your ability to follow prescribing guidelines?	
Do you know about the World Health Organization's Access, Watch, Reserve (AWaRe) antibiotic classification?	
If YES, does your facility consider the World Health Organization's AWaRe antibiotic guidance when prescribing antibiotics?	
If above answer is NO, please explain why not.	
What factors influence antibiotic prescribing at your facility? Can you list these?	
What are the challenges you face when prescribing antibiotics at your facility? Can you list these?	
Briefly describe how the above challenges influence the choice of antibiotics prescribed.	
Do patients expect or request antibiotics for acute respiratory infections?	
How do you explain to patients when antibiotics are not needed?	
Are there any non-clinical factors that influence your decision to prescribe antibiotics (e.g., fear of complications, patient satisfaction, time constraints)?	

Do you experience antibiotic supply challenges at your facility?	
If YES, are there any medicine shortages that affect your antibiotic prescribing decisions?	
What support or resources would help you improve antibiotic prescribing?	
Is antibiotic prescribing reviewed or audited at your facility?	
Are these audits paper based or undertaken with the help of electronic systems?	
Who is responsible for the audit or review at your facility?	
When was the last audit or review conducted at your facility?	
Have you received feedback on the audit? If yes, please describe the outcome of the audit and what activities are generally undertaken following the dissemination of the findings?	
What is your understanding of quality indicators?	
Have you used quality indicators to assess the quality of antibiotic prescribing at your facility?	
If above answer is YES, please describe the indicators that you have used.	

Have you assessed the quality of antibiotic prescribing in your facility within the last 12 months?	Yes	No
If **YES**, please explain how and what were the findings.		
If **NO**, provide reasons why it has not been done.		
**Quality indicators to assess the appropriateness of antibiotic prescribing in primary healthcare**		
**Explain the following to the participant:**		

The aim of this project is to develop and test quality indicators that can be used in future antimicrobial stewardship programmes, to assess and improve the appropriateness of antibiotic prescribing in public sector primary healthcare facilities in South Africa. The project also aims to assess the feasibility of a new easy-to-use Application (App) that can help healthcare professionals in primary healthcare to regularly track their prescribing of antibiotics for patients presenting with acute symptoms of infection. The quality indicators have been developed based on global indicators and on data collected from the multiple point prevalence studies that have been conducted at your facility to provide an overview of how antibiotics are prescribed and used at the primary healthcare level. I would like to hear your opinion about the implementation of quality indicators focused on assessing the quality of antibiotic prescribing, should they be implemented at the clinic.

Please indicate how much you agree or disagree with the following statements

The implementation of a dedicated App for antibiotic prescribing would be beneficial for tracking antibiotic prescribing patterns						
Strongly Disagree	Moderately Disagree	Slightly Disagree	Neutral	Slightly Agree	Moderately Agree	Strongly Agree
The implementation of quality indicators to assess antibiotic prescribing would be beneficial for the facility						
Strongly Disagree	Moderately Disagree	Slightly Disagree	Neutral	Slightly Agree	Moderately Agree	Strongly Agree
The implementation of the quality indicators will improve adherence to antibiotic guidelines						
Strongly Disagree	Moderately Disagree	Slightly Disagree	Neutral	Slightly Agree	Moderately Agree	Strongly Agree
The implementation of the quality indicators will negatively impact prescribing practices at the facility						
Strongly Disagree	Moderately Disagree	Slightly Disagree	Neutral	Slightly Agree	Moderately Agree	Strongly Agree

a) Please provide any recommendations that you have about the use and implementation of quality indicators for antibiotic prescribing and use at your facility.

b) Do you have any further comments or questions?

THANK YOU for your time and participation

Supplementary Figure S1: Inclusion and exclusion criteria for the PPS and clinimetric assessment.

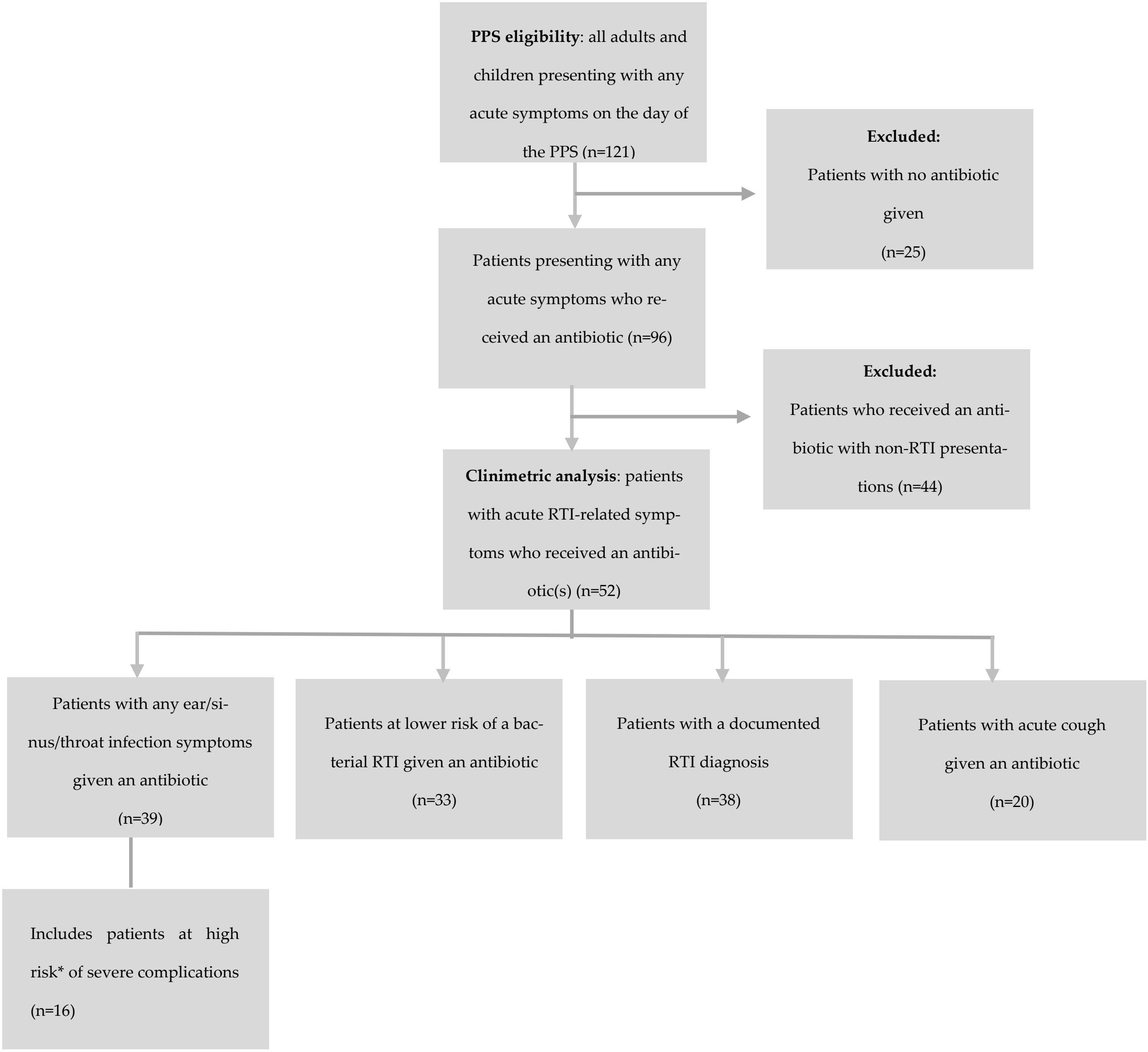

*High risk considered the documented bacterial infection diagnosis; the severity of symptoms e.g., fever and the risk of complications due to comorbidities including immunosuppression, patients living with human immunodeficiency virus (HIV) or patients with lung/heart/liver/kidney disease. In addition, the risk of rheumatic fever for pharyngitis, bilateral otitis in children below 2 years for otitis media as described in the WHO AWaRe antibiotic book was considered [29,43]; PPS = point prevalence survey; RTI= respiratory tract infection.
